# Early health technology assessment: current and future perspectives from a health technology assessment agency

**DOI:** 10.1017/S0266462325100482

**Published:** 2025-09-17

**Authors:** David Jarrom

**Affiliations:** Health Technology Wales, Velindre University NHS Trust, Cardiff, UK

**Keywords:** technology assessment, terminology as topic, value-based health care, decision making, translational research, biomedical

## Abstract

Health technology assessment (HTA) can occur at different stages of a technology’s lifecycle. In the accompanying paper, Grutters and colleagues present a consensus definition of “early HTA” as a health technology assessment conducted to inform decisions about subsequent development, research, and/or investment by explicitly evaluating the potential value of a conceptual or actual health technology. Early HTA is particularly relevant to non-medicine technologies, which are often developed more iteratively than medicines. This article explores some of the ways in which early HTA is already being conducted on non-medicine technologies in the United Kingdom, as well as future perspectives and possible challenges in using early HTA.

Health technology assessment (HTA) of medicines and non-medicine technologies uses similar methodologies, but compared to assessing medicines, the common challenges encountered when assessing non-medicine technologies differ. Ongoing evidence generation is more likely to be needed and more likely to mean that evidence-informed recommendations change over time. Iteration and development of the technology itself is also common, meaning that over time, its possible effectiveness and the use cases it supports may change. There is a growing recognition that HTA needs to consider the value of technologies at varying points in their development, rather than at one point in time (1;2). This “lifecycle approach” to HTA is particularly applicable to non-medicines technologies.

Early HTA, as defined by Grutters and colleagues (3), is part of the solution to some of these challenges. A range of different activities and processes can fall within the definition of early HTA. Technologies may have potential value to health and care systems, but do not yet have sufficient evidence to conclusively demonstrate that they are clinically and cost-effective. In these scenarios, early HTA can enable adopters to test and use the technologies in limited or controlled circumstances, such as for a time-limited period, or in the context of evidence generation. By highlighting what is required to enable more robust decision-making, early HTA can also ensure technology developers prioritize their resources towards generating suitable evidence. HTA is time and resource-intensive, and early HTA can provide a pragmatic alternative to this. For example, early HTA may focus only on some of the domains that would be considered in a fuller HTA, or use more pragmatic, less resource-intensive methods (while acknowledging any limitations or caveats this approach introduces). A more comprehensive HTA may then be carried out after production of evidence that fills a research gap previously highlighted by early HTA, or where early HTA indicates that a more detailed assessment is needed to rigorously judge the value of a technology.

Health Technology Wales (HTW) is a national health technology assessment body working to improve the quality of health and social care. HTW issues guidance on the use of selected technologies to health and social care providers in Wales. This guidance is underpinned by HTA methods (4).

Several steps within HTW’s current appraisal process fit within the definition of early HTA. For each technology potentially suitable for HTA, we produce a Topic Exploration Report (TER). These briefly summarize the characteristics of the technology being considered and give a high-level summary of the evidence available. TERs are used to inform decisions on whether the topic warrants further appraisal by HTW, but are also published as a standalone report, regardless of whether subsequent appraisal takes place. Although they do not make specific recommendations about a technology, TERs often highlight gaps in the evidence or further areas to target in a technology’s development, such as the need to define where in a care pathway a technology could offer the most benefit. HTW produces approximately 50 TERs per year (5). The effort that goes into early HTA needs to be proportionate, so these reports are based on a high-level scan of the literature, rather than using systematic search methods (4).

If a topic is selected for the appraisal work program, an Evidence Appraisal Report will be developed. This is used to inform guidance on the clinical and cost effectiveness of a technology. HTW’s Evidence Appraisal Reports and Guidance are produced on technologies that are relatively late in their life cycle: as a minimum, some evidence comparing the technology to current care needs to be available, and the technology must have relevant regulatory approval. But this work can still fall within the definition of early HTA. Examples would be cases where an Evidence Appraisal Report finds critical gaps or uncertainties about a technology’s clinical and/or cost effectiveness. Here, HTW’s guidance is unlikely to recommend widespread adoption, but specific recommendations are made for further research (6;7). HTW refers any research recommendations to research funders to highlight these priority evidence gaps, and to reduce barriers to this research being conducted. When future evidence generation addresses the gaps in the evidence highlighted by HTW’s guidance, technologies can be re-assessed and guidance updated. HTW has also used early HTA to inform the timing of fuller HTA by, for example, searching for and summarizing ongoing research in TERs. Where ongoing research is likely to be pivotal for decision making, HTW has deferred fuller HTA until such time as this evidence is available. Whether early HTA is based on a TER or Evidence Appraisal Report it also provides clearer signaling to developers about the evidence requirements of HTA.

Within the United Kingdom as a whole, the National Institute of Health and Care Excellence (NICE) has proposed changes to how they assesses non-medicines technologies (referred to by NICE as “Healthtech”). This utilizes the lifecycle approach and includes steps that meet the definition of early HTA. In 2022, NICE began a pilot of Early Value Assessments (EVAs) (8). These use HTA with the aim of providing quicker access to promising health technologies that address national needs. Technologies evaluated during the pilot had some evidence available, but often fundamental gaps in the evidence remained. For EVAs, NICE’s committees are not expected to make fully informed judgments about whether a technology is clinically or cost-effective. Instead, they focus on whether it is plausible that the benefits of the technology to patients, careers, and the system outweigh any harms. Where this is deemed to be the case, committees conditionally recommend technologies through this route. More evidence must be generated for any technology recommended through EVA. Evidence gaps are highlighted clearly in NICE’s resulting guidance recommendations, regardless of whether the technology is recommended or not recommended (9;10). Each EVA includes an evidence generation plan that makes specific recommendations about what evidence is needed to support future NICE guidance. NICE assesses the feasibility of this evidence generation with the aim of ensuring its recommendations are realistic and to maximize the chance of evidence generation being successful (11).

In early 2025, NICE set out its intention to make EVA a mainstream part of Healthtech evaluation (11). EVA will be referred to as “early use” in the future, and will apply only to non-medicine technologies; HTA of medicines at NICE will not change. This exemplifies the increasing importance of early HTA in the appraisal of non-medicines technologies specifically.

Some uncertainties or challenges remain with NICE’s proposed early use pathway. It is unclear at this stage how technologies will be selected as appropriate for consideration for early use as opposed to routine use. At the end of the evidence generation period, the evidence produced may not show that the technologies are clinically and cost-effective, or suitable evidence may not have been produced at all. In this scenario, the implications of withdrawing guidance that initially recommended the conditional use of a technology will need to be carefully considered. NICE’s proposed approach is iterative, and the evaluation of technologies more than once in their lifecycle may become more common. This could increase the overall resource needed for evaluation of non-medicine technologies, or reduce the number of non-medicine technologies NICE can appraise. Carefully considered prioritization of technologies for appraisal may be required.

Proponents of early HTA might argue that better reimbursement decisions during full HTA will be taken where early HTA has been performed first. Research in this area is currently sparse and in its infancy. Scientific advice has been shown to reduce the time to decision-making in subsequent full HTA (12;13). This research only covered medicines, and focuses exclusively on scientific advice or early dialogue, rather than core activity by HTA bodies that falls under the definition of early HTA. To the best of my knowledge, there has been no equivalent research on early HTA in health tech. The changes to non-medicines HTA in the United Kingdom do represent opportunities to carry out such research. Assessment of technologies through NICE’s healthtech program, and the subsequent evidence generation plans produced as part of these, present an opportunity to follow technologies up through their lifecycle. This could be done, for example, by research into the proportion of recommendations that change at the end of the early use and after evidence generation is completed. HTW also periodically re-assess technologies, and this also presents an opportunity to explore whether developers respond to research recommendations made by HTW, how frequently re-assessment occurs, and how frequently this results in changes to guidance. Wherever early HTA is studied, it is also important to seek the views of developers and other stakeholders through qualitative research.

What should HTA agencies and others do to make the best use of early HTA? The use of one consistent definition for this element of HTA is welcomed, and HTA agencies, journal editors, and other research organizations should heed the working group’s advice to use this term consistently in their HTA processes and in the wider literature. It is important that HTA agencies clearly publicize and signal evidence gaps where early HTA reveals these, and that incentives exist to carry out research to fill these evidence gaps. As the working group notes, some types of early HTA are not published at all (3). Wherever possible, findings from early HTA should be in the public domain and disseminated. This has the potential to reduce research waste and avoid the irreversible uptake of technologies that have not yet been conclusively shown to be clinically and cost-effective. Findings of early HTA are less likely to support broad use of a technology, because technologies are being assessed earlier in their lifecycle. Such recommendations can be more challenging for stakeholders to understand and accept. So, it will be important to communicate the intentions of early HTA to developers and other stakeholders clearly and explain to them how early HTA can improve the prospects of long-term adoption of a technology.

## References

[1] Pichler FB, Boysen M, Mittmann N, et al. Lifecycle HTA: promising applications and a framework for implementation. An HTAi global policy forum task force report. Int J Technol Assess Health Care. 2024;40(1):e50. 10.1017/S0266462324000187 38727087 PMC11569898

[2] Trowman R, Migliore A, Ollendorf DA. Health technology assessment 2025 and beyond: lifecycle approaches to promote engagement and efficiency in health technology assessment. Int J Technol Assess Health Care. 2023;39(1):e15. 10.1017/S0266462323000090 36815310 PMC11574536

[3] Grutters J, Bouttell J, Absrishami P, et al. Defining early health technology assessment: building consensus using Delphi technique. Int J Technol Assess Health Care. 2025. 10.1017/S0266462325100123 PMC12178750 40452233

[4] Health Technology Wales. Appraisal process guide. 2024. Available at: https://healthtechnology.wales/wp-content/uploads/Appraisal-Process-Guide.pdf [Accessed May 2025].

[5] Health Technology Wales. Reports and Guidance. 2025. Available at: https://healthtechnology.wales/wp-content/uploads/Appraisal-Process-Guide.pdf [Accessed May 2025].

[6] Health Technology Wales. Robot-assisted benign gynaecological surgery. 2024. Available at: https://healthtechnology.wales/reports-guidance/robot-assisted-benign-gynaecological-surgery/ [Accessed May 2025].

[7] Health Technology Wales. Artificial intelligence assisted tools for prostate cancer diagnosis using whole slide biopsy images. 2024. Available at: https://healthtechnology.wales/reports-guidance/artificial-intelligence-assisted-tools-for-prostate-cancer-diagnosis-using-whole-slide-biopsy-images/ [Accessed May 2025].

[8] National Institute of Health and Care Excellence. Early value assessment interim statement. Process and methods guide PGM39. 2024. Available at: https://www.nice.org.uk/process/pmg39 [Accessed May 2025].

[9] National Institute of Health and Care Excellence. Artificial intelligence-derived software to analyse chest X-rays for suspected lung cancer in primary care referrals: early value assessment. Health technology evaluation HTE12. 2023. Available at: https://www.nice.org.uk/guidance/hte12 [Accessed May 2025].

[10] National Institute of Health and Care Excellence. Digital technologies to support self-management of COPD: early value assessment. Health technology evaluation HTE19. 2024. Available at: https://www.nice.org.uk/guidance/hte19 [Accessed May 2025].

[11] National Institute of Health and Care Excellence. NICE HealthTech programme manual. Draft consultation document. 2025. Available at: https://www.nice.org.uk/guidance/indevelopment/gid-pmg10010 [Accessed May 2025].

[12] Maignen F, Kusel J. Assessing the value of Nice HTA scientific advice on the products appraised by NICE: a retrospective 10 year study. Value Health. 2020;23:S530. 10.1016/j.jval.2020.08.752

[13] Wang Y, Mccracken F, Landels H, Kusel J. The value of NICE scientific advice. ISPOR Europe; 2024. 10.1016/j.jval.2024.10.070

